# Supplementary material for: Polyamine Metabolism in Scots Pine Embryogenic Cells under Potassium Deficiency
Source: Cells. 2021 May 18;10(5):1244. doi: 10.3390/cells10051244 (PMC8158353; doi:10.3390/cells10051244)
Supplement: Supplementary file 1 [file cells-10-01244-s001.zip › cells-1186457-supplementary.pdf]

Polyamine metabolism of Scots pine under potassium deficiency

Muilu-Mäkelä Riina, Vuosku Jaana, Hely Häggman and Tytti Sarjala

Supplementary Data

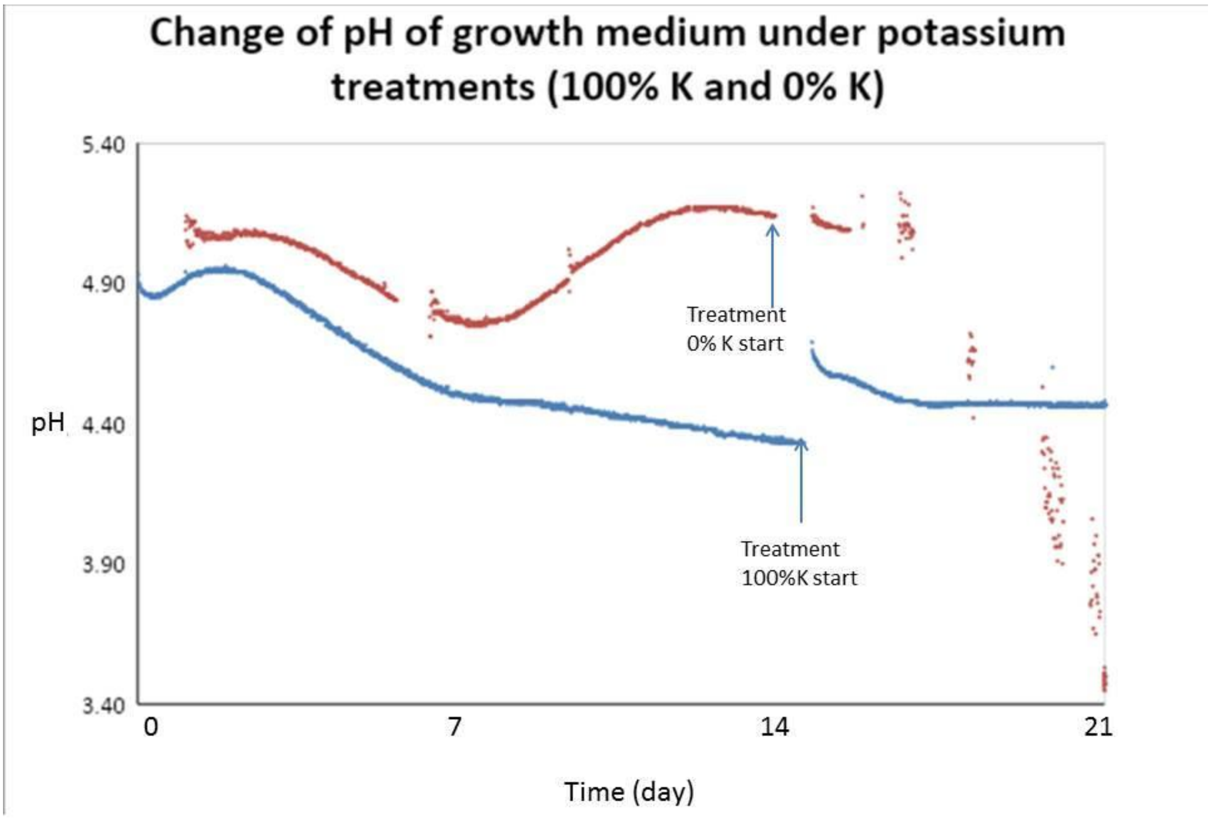

Supplemetary Figure S1. Change of pH of growth medium in two potassium treated bottles (100% K (indicated with blue) and 0% K (indicated with red)) during the experiment in the SENBIT system. In 0% potassium bottles pH decreased drastically from 5.1 to 3.4., whereas pH was over 4.5 in control bottles (100% K) throughout the experiment.

Supplementary Table S1. Modifications of DCR medium for potassium deficiency treatments. 100% K treatment was normal DCR potassium content (4.6 mmol/l), whereas in 0% K-treatment potassium was replaced by $(NH_4)_2HPO_4$. Concentrations of nitrogen (N) and phosphorus (P) were equal in all treatments.

Compound	DCR medium (100% K)	K 0%	K 150%
KNO_3	3,3629 mmol/l	0 mmol/l	5,0444 mmol/l
NH_4NO_3	5 mmol/l	5,429 mmol/l	5 mmol/l
$Ca(NO_3)_2 \bullet 4H_2O$	2,3542 mmol/l	2,3542 mmol/l	2,3542 mmol/l
$MnSO_4 \bullet H_2O$	0,131945 mmol/l	0,131945 mmol/l	0,131945 mmol/l
$ZnSO_4 \bullet H_2O$	0,047919 mmol/l	0,047919 mmol/l	0,047919 mmol/l
$CuSO_4 \bullet H_2O$	1,001 x 10^{-3} mmol/l	1,001 x 10^{-3} mmol/l	1,001 x 10^{-3} mmol/l
$MgSO_4 \bullet 7H_2O$	1,501165 mmol/l	1,501165 mmol/l	1,501165 mmol/l
KI	4,999 x 10^{-3} mmol/l	4,999 x 10^{-3} mmol/l	4,999 x 10^{-3} mmol/l
$CoCl_2 \bullet 6H_2O$	0,105 x 10^{-3} mmol/l	0,105 x 10^{-3} mmol/l	0,105 x 10^{-3} mmol/l
$NiCl_2 \bullet 6H_2O$	0,105 x 10^{-3} mmol/l	0,105 x 10^{-3} mmol/l	0,105 x 10^{-3} mmol/l
$CaCl_2 \bullet 2H_2O$	0,578175 mmol/l	0,578175 mmol/l	0,578175 mmol/l
H_3BO_3	0,10027 mmol/l	0,10027 mmol/l	0,10027 mmol/l
$Na_2MoO_4 \bullet 2H_2O$	1,033 x 10^{-3} mmol/l	1,033 x 10^{-3} mmol/l	1,033 x 10^{-3} mmol/l
KH_2PO_4	1,2492 mmol/l	0 mmol/l	1,8738 mmol/l
Thiamine-HCl	1 mg/l	1 mg/l	1 mg/l
Pyridoksine-HCl	0,5 mg/l	0,5 mg/l	0,5 mg/l
Niconine acid	0,5 mg/l	0,5 mg/l	0,5 mg/l
Glysine	2 mg/l	2 mg/l	2 mg/l
NaFeEDTA	0,04 g/l	0,04 g/l	0,04 g/l
Myo-Inositol	0,2 g/l	0,2 g/l	0,2 g/l
BAP	0,5 mg/l	0,5 mg/l	0,5 mg/l
2,4-D	2 mg/l	2 mg/l	2 mg/l
Casein hydrolysate	0,5 g/l	0,5 g/l	0,5 g/l
Saccharose	30 g/l	30 g/l	30 g/l
L-glutamiini $C_5H_{10}N_2O_3$	0,25 g/l	0,25 g/l	0,25 g/l
Phytagel (only in solid growth medium)	2,5 g/l	0 g/l	0 g/l
$(NH_4)_2HPO_4$		1,242 mmol/l	

Supplementary Table S2. PCR primers for Real-time PCR amplification of Scots pine PA metabolism (ADC, SPDS, ACL5, DAO and PAO), stress (CAT), programmed cell death (TAT-D) and cell division related (TAT-D) genes.

Gene	Forward primer	Reverse primer	PCR product size
ADC	5´-AGTCCGTGTGGCCTGTAATC-3´	5´-TGCACAGACACAACGTCAAA-3´	114
SPDS	5´-CCAACGTCCCATTAACCCTA-3´	5´-TGGCAAACAAAATGATGCTG-3´	106
ACL5	5´-ACTGCTCACATTCCGTCCT-3´	5´-TTCGCCTTTGATTCTCTGCT-3´	117
DAO	5´-AATGGGGAAGTTGGGAGTTC-3´	5´-CCCTCCTCAGTTTTCCAGTG-3´	102
PAO	5´-CGAAATTGCAGAACCTCCAC-3´	5´-CGGCCACGAACTACTCATCT-3´	95
CAT	5´-GGGAGGCAAACCTATGTGAA-3´	5´-TTGGTTGCATGACTGTGGTT-3´	110
RBR	5´-ACAGGAAGCAACCTCAGTGC-3´	5´-TCCACTGTCTCATGCCCTAA-3´	118
TAT-D	5´-TGGATGTTCCTTAAAGACAGTGG-3	5´-TCTCACAGTATGGAGCGTCTG-3	95

Supplementary Table S3. The effect of potassium treatments on proembryogenic cell mass growth, cellular viability and potassium concentration in the SENBIT system (linear model). Coefficients with 95% confidence intervals (CI) are presented. Coefficients with CIs not including zero are ststistically significant (indicated in bold).

	**FW (g)**		**Viability (abs)**		**potassium (mg/gDW)**	
treatment	Est.	CI 95%	Est.	CI 95%	Est.	CI 95%
intercept<br>K 100 %	10.8	8.9 –12.6	0.20	0.13–0.26	11.5	10.0–12.9
K 0 %	**-5.99**	**-8.63 – -3.34**	-0.01	-0.10–0.08	**-8.4**	**-11.1– -5.8**
K 50 %	**-3.84**	**-6.48 – -1.20**	-0.03	-0.12–0.06	**-4.9**	**-6.9– -2.8**
K 150 %	1.47	-1.17 – 4.11	**0.11**	**0.02–0.20**	2.0	-0.1–4.0

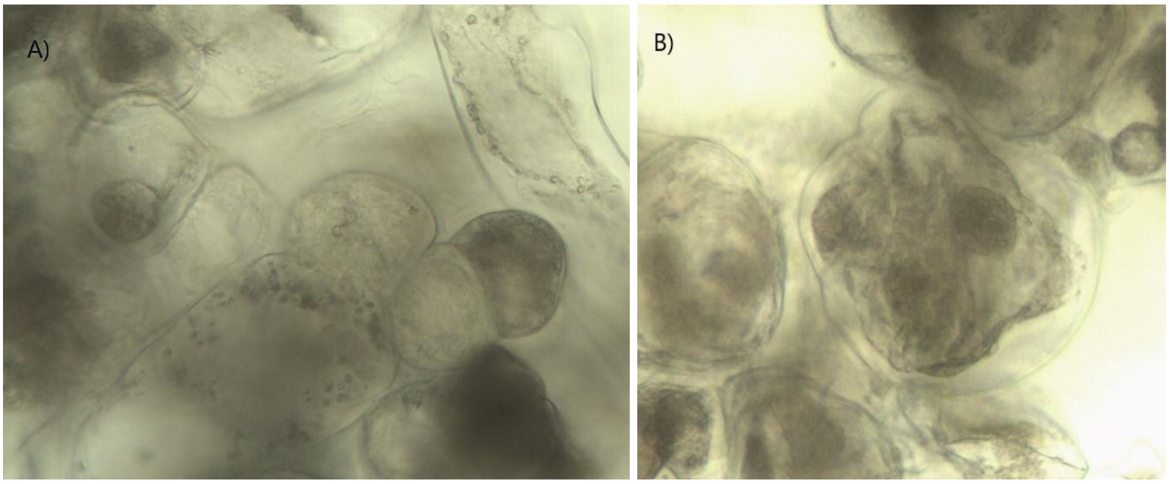

Supplementary Figure S2. Scots pine proembryogenic cells under A) 100% K (control) and B) 0% K treatments. Potassium deficiency decreased water content and tonus of the cells, which is seen as shrinkage of the cells when potassium content is 0%.

Supplementary Table S4. Mean and range of the dry weight (DW) per 1g fresh weight (FW) of the cell mass and moisture content of the cells under K treatments.

**Treatment**	**DW/gFW (cells)**		**MC% (cells)**	
	mean	range	mean	range
**0 %**	0.9	0.72–1	92.3	91.8–93.2
**50 %**	0.89	0.8–0.95	93.7	92.9–95.1
**100 %**	0.95	0.5–1.12	94.8	92.9–96.1
**150 %**	1.02	0.57–1.32	95.6	94.4–96.2

Supplementary Table S5. The effect of potassium treatments on baseline relative gene expression and estimated relative contrasts (with 95% confidence intervals CI) of key PA-enzyme genes (ADC, SPDS, ACL5, DAO and PAO) and three stress and programmed cell death related genes (CAT, RBR, TAT-D). Coefficients with CIs not including number one are statistically significant (indicated in bold).

	ADC		SPDS		ACL5		DAO		PAO		CAT		RBR		TAT-D	
treatment	Est.	CI 95%	Est.	CI 95%	Est.	CI 95%	Est.	CI 95%	Est.	CI 95%	Est.	CI 95%	Est.	CI 95%	Est.	CI 95%
Intercept K 100 %	0.97	0.75–1.26	0.99	0.85–1.17	0.86	0.54–1.37	0.95	0.71–1.26	0.97	0.59–1.60	0.99	0.78–1.26	1.40	1.09–1.78	1.55	1.21–1.98
K 0 %	1.31	0.90–1.89	**0.67**	**0.54–0.84**	**0.34**	**0.17–0.65**	0.82	0.55–1.24	**0.51**	**0.26–0.99**	**0.65**	**0.46–0.91**	**0.71**	**0.50–0.99**	**0.64**	**0.45–0.90**
K 50 %	1.36	0.94–1.97	0.93	0.74–1.17	**0.48**	**0.25–0.92**	0.93	0.62–1.40	**0.47**	**0.24–0.91**	0.77	0.55–1.08	0.76	0.54–1.08	**0.71**	**0.50–1.00**
K 150 %	0.93	0.64–1.35	0.93	0.74–1.16	1.12	0.58–2.18	0.86	0.57–1.28	1.15	0.59–2.24	**1.37**	**0.98–1.93**	0.94	0.67–1.33	0.95	0.67–1.35

Supplementary Table S6. The effect of potassium treatments on baseline relative polyamine concentrations and estimated relative contrasts (with 95% confidence intervals CI)**.** Coefficients with CIs not including number one are statistically significant (indicated in bold).

	**freePut**		**freeSpd**		**freeSpm**	
treatment	Est.	CI 95%	Est.	CI 95%	Est.	CI 95%
intercept K 100 %	366	308–434	105	85–130	23.18	1.73–2.94
K 0 %	**2.83**	**2.22–3.60**	1.31	0.97–1.77	1.12	0.74–1.70
K 50 %	**1.55**	**1.22–1.97**	1.05	0.78–1.42	0.99	0.65–1.50
K 150 %	**0.80**	**0.63–1.02**	1.07	0.79–1.44	1.07	0.71–1.63

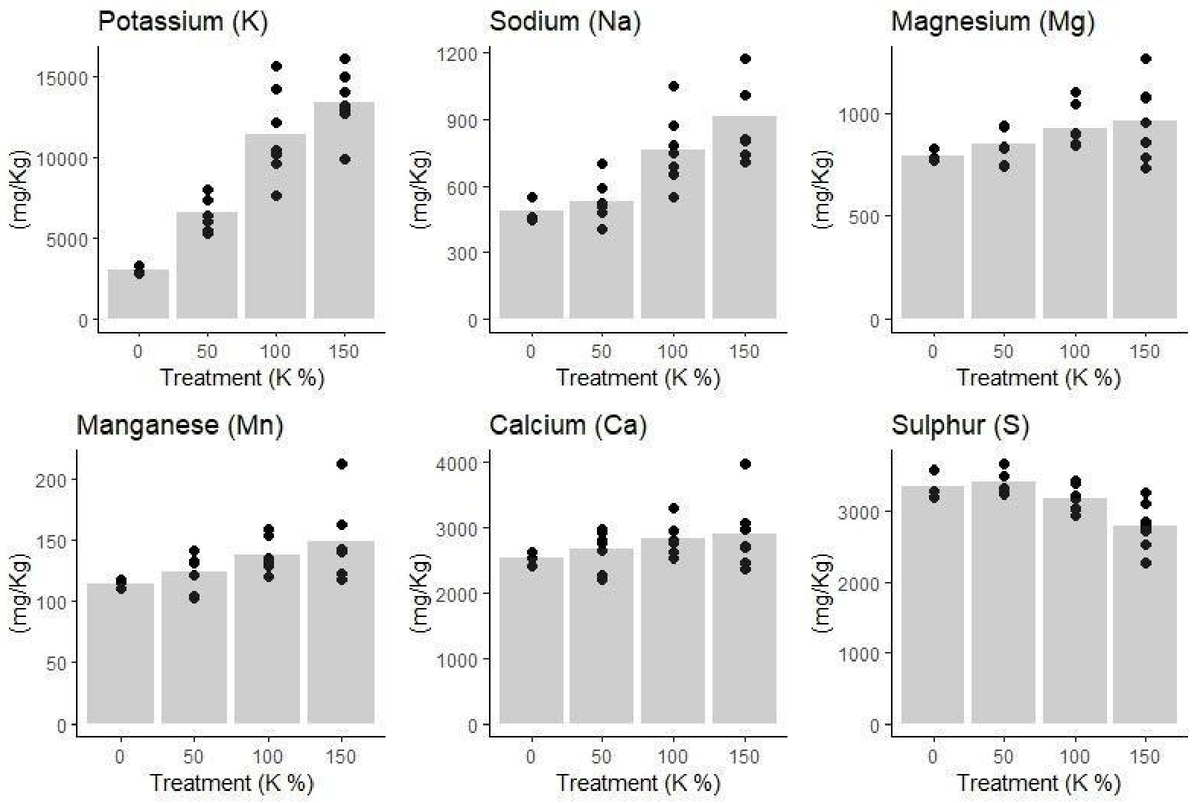

Supplementary Figure S3. Concentrations of potassium (K), sodium (Na), magnesium (Mg), manganese (Mn), calcium (Ca) and sulphur (S) in potassium treated cells.

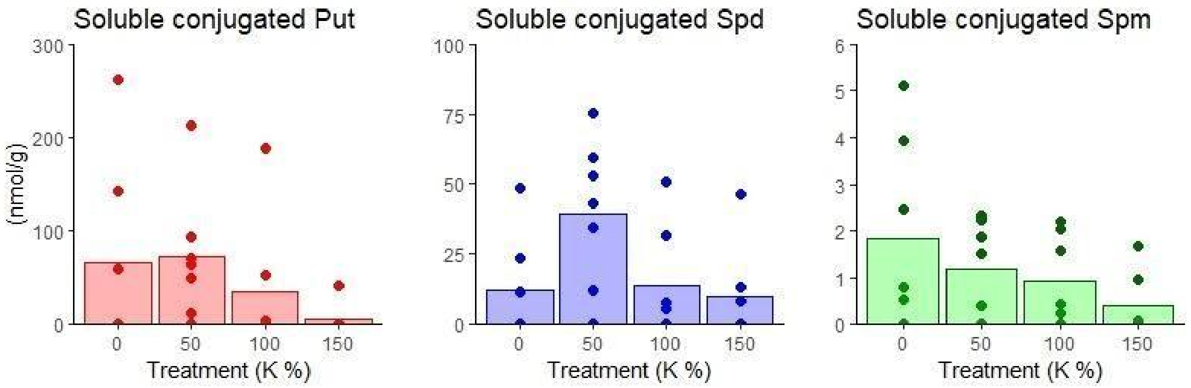

Supplementary Figure S4. Potassium treatment did not affect the content of soluble conjugated PAs, except probably soluble conjugated Put that was detected only from two out of seven bottles under potassium excess (K 150%).
